# Effects of Age and Working Memory Load on Syntactic Processing: An Event-Related Potential Study

**DOI:** 10.3389/fnhum.2018.00185

**Published:** 2018-05-04

**Authors:** Graciela C. Alatorre-Cruz, Juan Silva-Pereyra, Thalía Fernández, Mario A. Rodríguez-Camacho, Susana A. Castro-Chavira, Javier Sanchez-Lopez

**Affiliations:** ^1^Proyecto de Neurociencias, Facultad de Estudios Superiores Iztacala, Universidad Nacional Autónoma de México, Mexico City, Mexico; ^2^Laboratorio de Psicofisiología, Instituto de Neurobiología, Universidad Nacional Autónoma de México, Juriquilla, Mexico; ^3^Department of Psychology, Faculty of Health Sciences, University of Tromsø, Tromsø, Norway; ^4^Department of Neurosciences, Biomedicine and Movement Sciences, University of Verona, Verona, Italy

**Keywords:** working memory, normal aging, syntactic processing, LAN, P600a, P600b

## Abstract

Cognitive changes in aging include working memory (WM) decline, which may hamper language comprehension. An increase in WM demands in older adults would probably provoke a poorer sentence processing performance in this age group. A way to increase the WM load is to separate two lexical units in an agreement relation (i.e., adjective and noun), in a given sentence. To test this hypothesis, event-related potentials (ERPs) were collected from Spanish speakers (30 older adults, mean age = 66.06 years old; and 30 young adults, mean age = 25.7 years old) who read sentences to detect grammatical errors. The sentences varied with regard to (1) the gender agreement of the noun and adjective, where the gender of the adjective either agreed or disagreed with the noun, and (2) the WM load (i.e., the number of words between the noun and adjective in the sentence). No significant behavioral differences between groups were observed in the accuracy of the response, but older adults showed longer reaction times regardless of WM load condition. Compared with young participants, older adults showed a different pattern of ERP components characterized by smaller amplitudes of LAN, P600a, and P600b effects when the WM load was increased. A smaller LAN effect probably reflects greater difficulties in processing the morpho-syntactic features of the sentence, while smaller P600a and P600b effects could be related to difficulties in recovering and mapping all sentence constituents. We concluded that the ERP pattern in older adults showed subtle problems in syntactic processing when the WM load was increased, which was not sufficient to affect response accuracy but was only observed to result in a longer reaction time.

## Introduction

Aging results in cognitive changes, for instance, deterioration in working memory (WM) (Park et al., 2002). WM is a cognitive system that holds the available information while manipulating it for different cognitive tasks (Baddeley, 2003). There are various positions about how the limitations of this system may affect language. Just and Carpenter (1992) proposed that verbal WM has a limited capacity and requires storing linguistic information as it is processed. Thus, an increase in the complexity or length of the sentence may affect sentence comprehension because the information in feature bundles or chunks (i.e., partial representations of linguistic constituents of information; Lewis et al., 2006) decays in WM. Nevertheless, other authors propose that each language process is supported by a different WM resource, which can prevent information decay (Waters and Caplan, 1996; Jackendoff, 2007).

Aging studies have supported the idea that the increases in demands of WM affect language processing, since older adults show behavioral difficulties in comprehension of embedded syntactic structures (Kemper, 1986, 1987; Kemper et al., 1990, 2001). Such difficulties can be explained as a failure to maintain linguistic elements in WM (King and Kutas, 1995). Syntactic processing involves agreement rules between lexical units (e.g., in the phrase “She works,” the pronoun “She” inherits the number agreement to the verb “work” and it is added a suffix “s”). Among the features used to compute agreement is the marking of gender, number, person or case (Chomsky, 1995). In the Minimalist Program (Chomsky, 2001), agreement entails copying of these features from one lexical unit to another; the controller is the element from which grammatical information originates (e.g., the pronoun), and the target is the element that inherits the information (e.g., the verb). It has been suggested that a larger number of lexical units—without agreement features—between the controller and the target make language comprehension more difficult due to the subjects having to maintain in WM the controller’s features until the target is found (Van Dyke, 2007; Van Dyke and Johns, 2012).

One way to study WM is through the use of event-related potentials (ERPs). ERPs are voltage fluctuations of brain electrical activity recorded over the scalp time-locked to external or internal stimuli. Their high temporal resolution allows fine sequential analysis of the cognitive processes involved in a task. These waveforms are analyzed according to their amplitude, latency and topography distribution of voltage over the scalp (Luck, 2005). The amplitude reflects the brain electrical activity when a specific computational operation is performed (Osterhout et al., 2006), and meanwhile the latency is the point at which the voltage reaches a local maximum or minimum (Luck, 2005).

Event-related potential studies have evidenced the effect of increasing WM load demands on syntactic processing in young adults. In these studies, WM load was manipulated by length (i.e., the total number of words in a sentence between two lexical units with shared agreement features) or by syntactic complexity (i.e., the number of phrases syntactically analyzed). In particular, a high WM load is associated with increases in reaction times (RTs) and poorer sentence comprehension (King and Just, 1991; Vos et al., 2001). These studies have shown changes in the electrophysiological pattern of ERP as the WM load is increased. Vos et al. (2001) described greater amplitude of a left-anterior negativity (LAN component) when the syntactic complexity (i.e., embedded vs. conjoined sentences) was increased. LAN occurs between 300 and 500 ms after stimuli and, in language processing tasks, is modulated by morpho-syntactic processing (Friederici, 1995, 2002; Osterhout and Holcomb, 1995; Osterhout and Mobley, 1995; Bornkessel and Schlesewsky, 2006).

Event-related potential studies that manipulate WM load have also reported greater difficulties in agreement processing of verbal inflections that is reflected in a smaller P600 amplitude when syntactic complexity is increased (Gunter et al., 1997). A smaller or absent P600 effect has been observed in subject-verb agreement (i.e., amplitude differences of ERPs between disagreeing and agreeing conditions) when syntactic complexity was increased (i.e., relative sentences, Kolk et al., 2003). Greater processing cost in the construction of syntactic dependencies is reflected in longer P600b latencies when the sentence length (i.e., the distance of “wh” dependency) is manipulated (Vos et al., 2001; Phillips et al., 2005).

P600 is a positivity that peaks at approximately 600 ms with an onset at 500 ms and lasts for several hundreds of milliseconds over central sites on the scalp. It has been linked to syntactic processing (Osterhout and Mobley, 1995; McKinnon and Osterhout, 1996; Osterhout et al., 1996, 1997, 2002; Osterhout and Nicol, 1999; Kaan, 2002) and syntactic reanalysis (Friederici, 2002). According to some authors (Barber and Carreiras, 2005; Silva-Pereyra and Carreiras, 2007; Molinaro et al., 2008), this reanalysis process can be separated into two consecutive processing steps. The first step integrates all of the information associated with the processed-critical word concerning the previous sentence context (Kaan et al., 2000; Friederici et al., 2001; Barber and Carreiras, 2005). This step is reflected by a P600a (i.e., between 500 and 700 ms) that is located at anterior medial sites. In the second step, a generalized mapping of sentences (i.e., evaluation of well-formedness; Bornkessel and Schlesewsky, 2006) may be performed. Then, a P600b (i.e., 700–900 ms) appears with a posterior distribution (Friederici et al., 2001; Barber and Carreiras, 2005; Bornkessel and Schlesewsky, 2006). Currently, these subcomponents of the P600 are poorly understood and are a topic of debate; however, this pattern of P600 “a” and “b” may be useful to show differences in brain activity associated with sentence reanalysis between two different populations.

In aging, language processing is associated with longer latencies and smaller amplitudes of many ERP components (Wlotko et al., 2010). Only one ERPs study has described age-related changes associated with syntactic processing, but it did not manipulate the WM load. This study explored the ERP changes during sentence comprehension when number agreement was manipulated. No differences were found in accuracy and time response between old and young adults (Kemmer et al., 2004). However, the results of this study showed a more asymmetric and frontal topographic distribution of the early P600 in older adults than in young participants.

Therefore, considering that older adults show (a) behavioral problems in sentence comprehension with increased syntactic complexity (Kemper, 1986, 1987; Kemper et al., 1990, 2001) and (b) ERP amplitude changes in syntactic processing compared with the young participants (Kemmer et al., 2004), this study aimed to assess the effect of WM load (i.e., syntactic complexity) and gender agreement on sentence processing as a function of age. We manipulated the WM load by varying the distance between the noun and adjective during gender agreement processing.

We expected that with a greater syntactic complexity (i.e., a high WM load condition), the elderly participants would show longer RTs and have fewer correct answers than young adults; however, when syntactic complexity was negligible, we did not expect to find behavioral differences between the groups. Given that a higher WM load imposes a greater cost associated with agreement processing, we expected that under a high WM load, older adults would show a greater cost in morpho-syntactic processing than young adults, and this might be reflected in greater amplitudes of the LAN component. We expected that elderly participants would show more problems in integrating all of the information associated with the previous sentence context and that this would be reflected in a smaller amplitude of the P600 component. We also hypothesized that older adults would show a greater processing cost during the generalized mapping of sentences, which could be observed in longer latencies of the P600b component.

## Materials and Methods

### Participants

Thirty older adults (mean age = 66.06 years old, *SD* = 4.9, range = 60–80 years old; 20 female) and 30 young adults (mean age = 25.7 years old, *SD* = 4.9, range = 19–35 years old; 17 female) participated in this study. All of them were ethnic Mexican, right-handed, native Spanish speakers and healthy (with no history of neurological or psychiatric disorders). Right-handedness was assessed with a brief Spanish version of the Edinburgh Handedness Inventory (Oldfield, 1971): laterality quotient (LQ) > +50. All subjects had at least 9 years of education (no differences between groups in educational attainment; older adults: mean = 16.8, *SD* = 6.2; young adults: mean = 18.1, *SD* = 3.0; *t*(58) = -1, *p* = 0.32). In addition, there was no significant sex difference between groups (older adults: 66% women, and young adults: 52.8%; χ^2^ = 1.1, *p* = 0.3).

The older adults were evaluated using the Global Deterioration Scale (GDS) (Reisberg et al., 1982). Only subjects with scores of 1 or 2, indicating the absence of cognitive decline, were included in this study. The Wechsler Adult Intelligence Scale in Spanish (WAIS-III, Wechsler, 2003) was administered to the participants. All subjects obtained scores above 90 on the full score of the WAIS-III. This scale has four indices scores: verbal comprehension index (VCI), working memory index (WMI), perceptual organization index (POI), and processing speed index (PSI). We assessed between groups differences per index. Four mixed two-way ANOVAs were performed considering group (older adults and young adults) as a between-subjects factor. VCI subscales (i.e., vocabulary, similarities, and information), WMI subscales (i.e., arithmetic, digit span, and letter-number sequencing), POI subscales (i.e., block design and matrix reasoning) and PSI subscales (i.e., digit symbol-coding and symbol search) were included as within-subjects factors for each ANOVA. No significant differences between groups were observed in VCI [Group: *F* < 1; group by VC: *F*(1,58) = 2.254, *p* = 0.117, $\eta_{p}^{2}$ = 0.037, ε = 0.873] or WMI [Group: *F* < 1; group by WM: *F*(1,58) = 1.577, *p* = 0.24, $\eta_{p}^{2}$ = 0.026, ε = 0.873] indices. However, in POI young adults (block design mean, *M* = 13.6, standard deviation, *SD* = 2.8; matrix reasoning *M* = 12.5, *SD* = 2.2) showed a better performance than older adults (block design *M* = 11.7, *SD* = 3.2; matrix reasoning *M* = 10.6, *SD* = 3.1) [Group: *F*(1,58) = 7.812, *p* = 0.007, $\eta_{p}^{2}$ = 0.119]. In the same way, in PSI, young adults (digit symbol-coding *M* = 13.9, *SD* = 2.2; symbol search *M* = 13.0, *SD* = 1.8) showed a better performance than older adults (digit symbol-coding *M* = 11.4, *SD* = 3.4; symbol search *M* = 11.2, *SD* = 2.4) [Group: *F*(1,58) = 13.613, *p* < 0.001, $\eta_{p}^{2}$ = 0.190].

To ensure that all participants (older adults and young adults) had a normal electroencephalogram (EEG), they were assessed using eye-closed resting state EEG. Quantitative EEG from 19 electrodes was fast-Fourier transformed to obtain cross-spectral matrices every 0.39 Hz. The absolute power (AP) with geometric power correction (Hernández et al., 1994) was calculated every 0.39 Hz. *Z*-scores for AP and relative power (RP) were calculated in four frequency bands: delta (1.5–3.5 Hz), theta (4–7.5 Hz), alpha (8–12.5 Hz), and beta (13–19.5 Hz), comparing subject measures with the norm measures (Valdés et al., 1990; Szava et al., 1994). *Z*-values lower than 1.96 were considered within the normal limits concerning the age group of every subject. Additionally, an expert neurophysiologist performed a visual inspection of the EEGs to exclude subjects with abnormal waves. All participants were informed of their rights and provided written informed consent for participation in the study. This research was carried out ethically and was approved by the Ethics Committee of the Instituto de Neurobiología at the Universidad Nacional Autónoma de México (Ethical Application Ref: INEU/SA/CB/109).

### Stimuli and Procedure

Nouns, verbs, and adjectives were selected from the LEXMEX corpus (Mexican computerized database of the Spanish language with word use frequency; Silva-Pereyra et al., 2014) according to their frequency. All words with more than 30 appearances/million were included. Nine hundred sentences of seven words each in Spanish were built, which were read and judged with respect to their appropriateness in their common use by 15 subjects (outside of the context of the ERP experiment). Two hundred and twenty sentences were selected for the experiment from those that all of these 15 participants considered to be well-formed. Eighty sentences comprised gender agreement and 80 sentences gender disagreement between the noun of the main clause and its adjective. Disagreement sentences were built changing the derivational morpheme of gender for the qualifying adjective, i.e., *rojo* – *roja* (red_Masculine_ – red_Feminine_, with the last morpheme indicating masculine or feminine, respectively) (see **Table 1**). The adjective expressed a characteristic of the main noun in the sentence, and all of the nouns designated inanimate objects (same proportion of genders). Eighty were sentences in the Agree condition, and 80 were sentences in the Disagree condition.

**Table 1:** Examples of stimuli presented to subjects.

WM Load	Gender agreement	Example
Low	Agree	La casa_Fem_ **amarilla**_Fem_ está en la colina
		[The **yellow** house is on the hill]
	Disagree	El carro_Masc_ **amarilla**_Fem_ está en la colina
		[The **yellow** car is on the hill]
High	Agree	La casa_Fem_ que está allá es **amarilla**_Fem_
		[The house over there is **yellow**]
	Disagree	El carro_Masc_ que está allá es **amarilla**_Fem_
		[The car over there is **yellow**]


**Nouns are underlined, and adjectives are typed in bold fonts. Fem, feminine; Masc, masculine.**

There was a “WM Load” factor (i.e., syntactic complexity: number of nodes parsed, Van Dyke and Johns, 2012) with two levels, low and high, and an “Agreement” factor with two levels, agree and disagree. Forty agree and 40 disagree sentences were low WM load sentences and forty agree and 40 disagree sentences were high WM load sentences where a clause was embedded within the noun-adjective agreement or disagreement. This clause was placed between lexical units with a dependent syntactic relationship (e.g., disagree/high WM load: El carro_Masculine_ que está allá es amarilla_Feminine_ [The car_Masculine_ over there is yellow_Feminine_], e.g., disagree/low WM load: El carro_Masculine_ amarilla_Feminine_ está en la colina [The yellow_Feminine_ car_Masculine_ is on the hill_Feminine_]). Sixty additional sentences were included as fillers, with 30 grammatical and 30 ungrammatical sentences. These sentences had the same syntactic structure, but different syntactic manipulation (i.e., number agreement).

The task was presented to subjects using STIM2 software (NeuroScan, CompuMedics, Charlotte, NC, United States) on a computer screen while subjects were seated at a distance of 70 cm from the screen. Subjects read the task’s instructions on the screen. They were instructed to read the whole sentence and only respond as efficiently and quickly as possible when the question marks appeared. The words in white were displayed at the center of the black screen; the type font was Arial and the size was 80. At the beginning of every sentence, a fixation cross was presented for 300 ms. Sentences were given one word at a time for 300 ms each with an inter-stimulus interval of 300 ms (i.e., the words were successively presented and disappeared after that, before the next word appeared). At the end of the sentence, two question marks appeared for 1500 ms. At that moment, subjects were required to answer whether the sentence was correct (grammatical) or not, pressing one of two buttons using their thumbs on a response box. One button was for “correct” sentences (gender/number agreement), and the other was for “incorrect” sentences (gender/number disagreement). Response buttons were counterbalanced among subjects. The task took 35 min; the subjects had three rest periods, one every 9 min.

### ERP Acquisition

The EEG was recorded using 32 silver electrodes embedded in an elastic cap (Electro-Cap International, Inc., Eaton, OH, United States), each referenced online to the left earlobe (A1). A2 was also recorded. The EEG was amplified with the NeuroScan SynAmps system (Scan 4.5 software; NeuroScan, CompuMedics, Charlotte, NC, United States) with a bandwidth of 0.1–100 Hz and was digitized at a 500 Hz sampling rate. The recordings were referenced offline to averaged earlobe signals. Electrode impedances were kept below 2 kΩ. The electrooculogram (EOG) was also recorded with electrodes located on the external canthus and the supraorbital ridge of the left eye. An EOG artifact correction method (Scan 4.5 software; NeuroScan, CompuMedics, Charlotte, NC, United States) was applied to the EEG data offline. Segments with artifacts were rejected.

### Data Analysis

#### Behavioral Data

For the statistical analysis of behavioral data, two mixed three-way ANOVAs were performed to analyze the RTs and percentages of correct answers. Group (young adults and older adults) was included as the between-subjects factor, and gender agreement (agree and disagree) and WM load (high and low) were included as within-subjects factors. Tukey’s honestly significant difference (HSD) method was performed for *post hoc* pairwise comparisons. Percentages of CA were transformed using the function {ARCSINE [Square Root (percentage/100)]} to ensure a normal distribution of the data (Zar, 1998).

#### ERP Data

The ERPs were computed offline using epochs of 200 ms pre-stimulus and 1000 ms post-stimulus per subject and per experimental condition (disagree/low WM load, disagree/high WM load, agree/low WM load, and agree high WM load). Given that adjectives in Spanish have a post-nominal position, the ERPs for the adjectives in each sentence were obtained. Averaged waveforms included only those trials with correct answers and with voltage changes lower than ±50 μV. Trials with artifacts due to eye movement, excessive muscle activity, or amplifier blocking were eliminated offline before averaging. Baseline correction was performed using the 200 ms pre-stimulus time window. The number of useful segments for the ERPs average was approximately 25, the same for both groups and all conditions. In older adults, 63% of the artifact-free segments were retained in the low WM load condition and 60.6% in the high WM load condition. Meanwhile, in young adults, 65% of the segments were retained in a low WM load and 61.5% in a high WM load condition.

**FIGURE 1:**
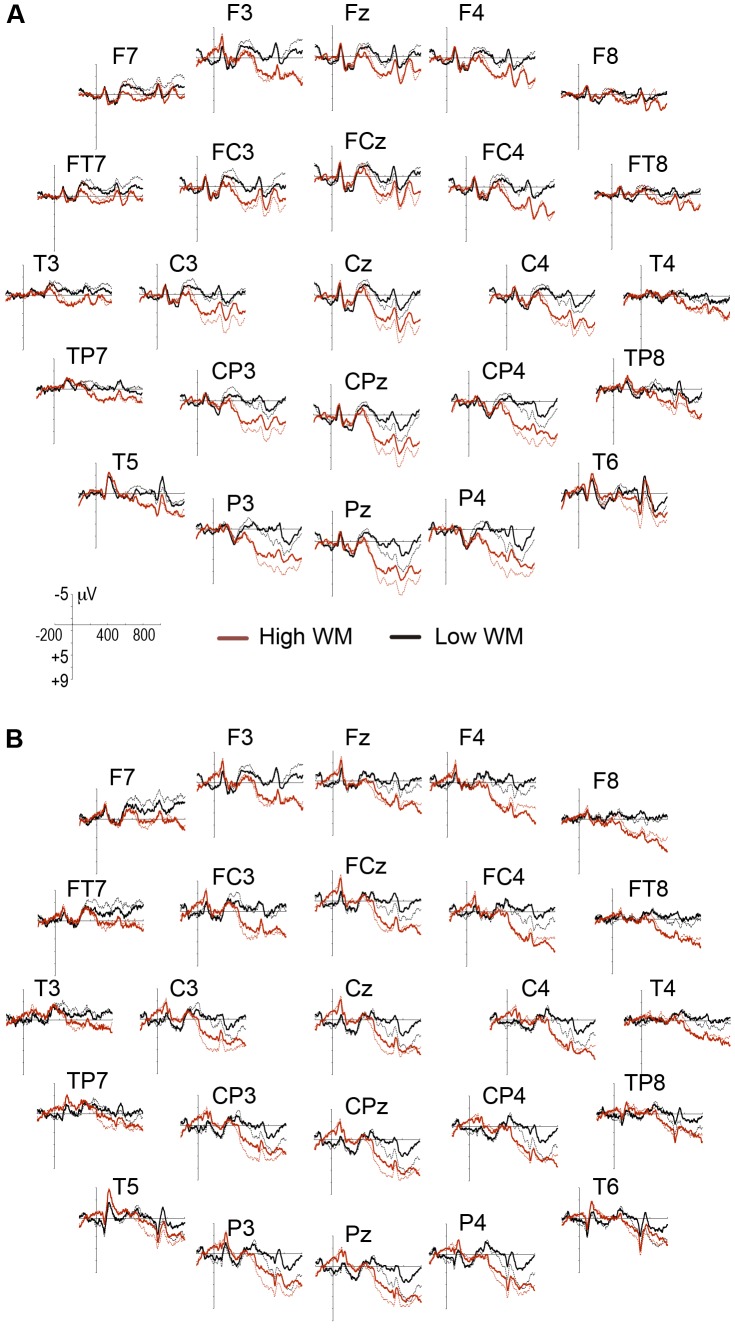
Event-related potentials (ERP) wave grand averages across 25 electrode sites of **(A)** young adults and **(B)** older adults. Black lines represent low working memory (WM) load and red lines represent high WM load. ERP responses to agree and disagree conditions are represented by the solid and dotted lines, respectively. The negative amplitude is plotted upwards.

Difference waves (i.e., ERPs of disagree condition minus ERPs of agree condition) to the critical adjective are presented in **Figure 1**. This figure shows a negative wave at 300–500 ms with maximal amplitudes at anterior sites (i.e., the LAN component), which was followed by two positive waves, the first at 500–700 ms (i.e., P600a) and the second at 800–1000 ms (i.e., P600b) with maximal amplitudes at central and posterior sites, respectively. This LAN-P600a–P600b pattern is similar to that previously reported in gender-agreement studies (Barber and Carreiras, 2005; Molinaro et al., 2011). To test the processing cost of increases in WM load (i.e., modulations in LAN and P600 amplitudes), mixed three-way ANOVAs were separately performed on the mean amplitude values for LAN, P600a, and P600b windows. In the LAN window, group was included as a between-subjects factor and WM load and anterior electrodes (F7, F3, Fz, F4, and F8) as within-subjects factors. In the P600a window, group was included as a between-subjects factor and WM load and central electrodes (C3, Cz, C4, CP3, CPz, and CP4) as within-subjects factors. In P600b, group was included as a between-subjects factor and WM load and posterior electrodes (P3, Pz, and P4) as within-subjects factors. The analyzed electrodes were selected from previous literature about the topographical location of these components (Molinaro et al., 2011). A mixed three-way ANOVA was performed on the latency data for the maximum amplitude within the range of P600b. Group was included as a between-subjects factor, and WM load and posterior electrodes were included as within-subjects factors. Tukey’s HSD was performed for *post hoc* pairwise comparisons.

For each group, a Pearson correlation test was performed to explore the relationship between behavioral performance (percentage of correct answers or RTs) and difference-wave amplitude of the ERPs (LAN: anterior electrodes; P600a: central electrodes; P600b: posterior electrodes). Additionally, behavioral performance was also correlated with P600b latency (posterior electrodes).

## Results

### Behavioral Results

**Table 2** summarizes the behavioral performance, showing RTs and percentage of correct answers for both groups. There were no differences between groups regarding the percentage of correct answers [Group: *F*(1,58) = 2.43, *p* = 0.13, $\eta_{p}^{2}$ = 0.04]. However, older adults were slower than young adults to give their response in all of the experimental conditions [Group: *F*(1,58) = 8.52, *p* = 0.005, $\eta_{p}^{2}$ = 0.13]. No significant interactions of WM load or gender agreement by group were observed in the percentage of correct answers or RTs analysis.

**Table 2:** Mean reaction times (RT) and percentage of correct answers and the corresponding standard deviations (SD) for both of the four experimental conditions.

		Young	Adults	Older	Adults
			RT ms		RT ms
		
WM load	Agreement	% CA (*SD*)	Mean (*SD*)	% CA (*SD*)	Mean (*SD*)
Low	Agree	67.8 (21.3)	391.7 (72.2)	73.9 (17.4)	447.2 (87.0)
	Disagree	66.0 (18.0)	368.5 (71.5)	75.7 (19.3)	428.9 (88.5)
					
High	Agree	68.6 (19.2)	407.1 (80.6)	73.1 (20.0)	472.0 (81.2)
	Disagree	68.8 (19.1)	395.3 (94.6)	76.4 (19.7)	450.1 (105.3)


### Electrophysiological Results

#### Amplitude Analysis of LAN Effect

**FIGURE 2:**
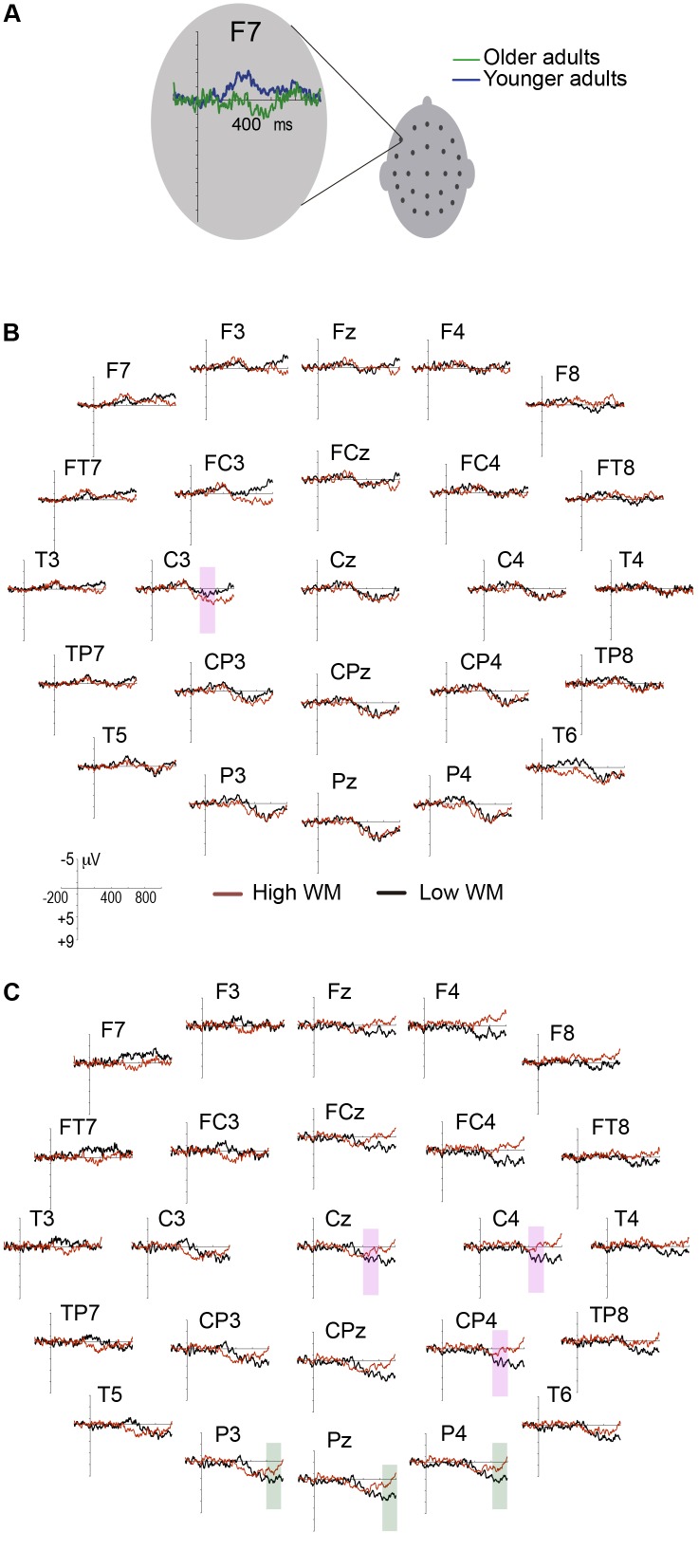
Difference wave of ERPs. **(A)** Amplitude significant difference between groups in the LAN effect at F7, **(B)** young adults, and **(C)** older adults. Magenta shaded boxes indicate significant differences between high and low WM load at the time window of P600a and orange shaded boxes indicate significant differences between high and low WM load at the time window of P600b. The negative amplitude is plotted upwards.

ANOVA results showed a significant group by WM load by anterior electrodes interaction [*F*(4,232) = 4.08, *p* = 0.01, $\eta_{p}^{2}$ = 0.07, ε = 0.69]. *Post hoc* tests showed significant differences between groups for the F7 site (see **Figure 2**). In the high WM load condition (MD = 1.95, *p* = 0.03) older adults displayed smaller amplitudes of the LAN effect than young adults did. Older adults showed (MD = 1.60, *p* = 0.03) a greater amplitude of the LAN effect in the low than in the high WM load condition; meanwhile, young adults did not show differences between the WM load conditions (see **Table 3**).

**Table 3:** Means and standard deviations (SD) in microvolts (μV) of ERP effects (difference waves).

			High WM load		Low WM load	
				
			Mean	*SD*	Mean	*SD*
**LAN**	F3	Older adults	0.18	2.60	-1.16	3.24
	F4		-0.60	2.75	0.52	2.93
	Fz		0.65	3.33	-0.35	3.38
	F7		0.41*	2.90	-1.18*	2.94
	F8		-0.57	2.90	-0.11	2.11
	F3	Younger adults	-1.31	3.55	-0.65	3.05
	F4		-0.82	2.60	-0.57	2.25
	Fz		-0.94	3.43	-0.36	2.60
	F7		-1.54*	3.70	-0.74	2.54
	F8		-0.84	2.79	-0.25	2.80
**P600a**	C3	Older adults	1.51	3.24	0.18	3.50
	C4		0.48*	3.80	1.01*	3.45
	Cz		1.23**	3.88	0.97**	3.63
	CP3		1.68	3.25	0.44	3.41
	CP4		0.84**	3.72	1.16**	3.35
	CPz		1.45	3.80	1.03	3.72
	C3	Young adults	1.55*	3.46	0.64*	2.29
	C4		1.05	2.84	0.81	2.50
	Cz		1.50	3.24	1.13	2.75
	CP3		1.44	3.25	0.66	2.40
	CP4		1.40	3.20	1.00	2.60
	CPz		1.71	3.40	1.30	2.75
**P600b**	P3	Older adults	0.79*	3.36	2.36*	4.17
	P4		0.70*	3.00	2.52*	3.92
	Pz		0.27*	3.49	2.40*	4.31
	P3	Young adults	1.65	3.41	1.20	3.52
	P4		1.46	4.00	1.92	3.25
	Pz		1.73	3.70	1.90	4.10


**^∗^p < 0.05, ^∗∗^p < 0.01. Post hoc results of comparison between high and low WM load.**

To know whether reduced LAN effects in the older group during the high WM condition was due to an increased amplitude of agree condition or a reduced amplitude of disagree condition, a three-way ANOVA was performed with the mean amplitude values of LAN component only for the group of older adults. WM load, Agreement, and anterior electrodes were included as within-subjects factors. A significant WM load by Agreement by anterior electrodes interaction [*F*(4,116) = 9.35, *p* < 0.01, $\eta_{p}^{2}$ = 0.24, ε = 0.61] was observed. *Post hoc* test showed that in disagree condition, there were smaller amplitudes in F3 (MD = -1.66, *p* = 0.02), Fz (MD = -1.47, *p* = 0.03) and F7 (MD = -1.58, *p* = 0.02) sites during high, as opposed to the low WM load condition. In contrast, no significant amplitude-differences between WM load conditions were observed in agree condition.

#### Amplitude Analysis of the P600a Effect

There was a significant group by WM load by central electrodes interaction [*F*(5,290) = 2.70, *p* = 0.05, $\eta_{p}^{2}$ = 0.04, ε = 0.6]. *Post hoc* tests did not show significant differences between groups, but differences between the WM load conditions for each group were observed. Older adults showed a smaller P600a amplitude effect in the high than in the low WM load from the C4 (MD = 2.01 μV, *p* = 0.03) and CP4 electrodes (MD = 2.61, *p* = 0.004); and greater amplitude at the Cz site (MD = -2.43, *p* = 0.006). Meanwhile, young adults displayed a greater P600a amplitude effect for the high WM load than for the low WM load condition at the C3 site (MD = -1.82, *p* = 0.05) (see **Table 3**).

#### The Amplitude and Latency Analysis of the P600b Effect

There was a significant group by WM load by posterior electrodes interaction for the amplitude analysis [*F*(2,116) = 3.70, *p* = 0.03, $\eta_{p}^{2}$ = 0.06, ε = 0.9]. *Post hoc* tests did not show significant differences between groups, but differences between WM load conditions were found only for older adults; a smaller amplitude effect in the high WM load condition than in the low WM load condition was observed (P3: MD = 2.09, *p* = 0.02; P4: MD = 1.73, *p* = 0.04; Pz: MD = 1.70, *p* = 0.04). There was no significant group by WM load by posterior electrodes interaction when latency analysis was performed (see **Table 3**).

### Correlations Between Behavioral Data and ERP Amplitude Effects

No significant correlations between behavioral performance and amplitude effects in the low WM load condition were observed. **Table 4** displays significant correlations in the high WM load condition. Regarding the percentage of correct answers in the agree condition, only the young adult’s group showed significant positive correlations with the LAN effect at Fz. In both agreement conditions, the percentage of correct answers was positively correlated with the P600a effect at almost all electrodes analyzed (see **Table 4**).

**Table 4:** Person’s correlations (rho) between behavioral performance and difference-wave amplitudes in High WM load condition.

		Older adults		Young adults	
			
High WM load condition		LAN	P600a	LAN	P600a
Percentage of correct answers	Agree			Fz (0.41)^∗^	C3 (0.50)^∗∗^
					C4 (0.45)^∗^
					Cz (0.42)^∗^
					CP3 (0.43)^∗^
					CP4 (0.45)^∗^
	Disagree				C4 (0.37)^∗^
					Cz (0.45)^∗^
					CP3 (0.44)^∗^
					CP4 (0.42)^∗^

RTs	Agree	F3 (-0.39)^∗^		F7 (-0.41)^∗^	C3 (-0.37)^∗^
		Fz (-0.39)^∗^			Cz (-0.37)^∗^
					CP3 (-0.37)^∗^
	Disagree	F3 (-0.42)^∗^	C3 (-0.40)^∗^	F7 (-0.41)^∗^	C3 (-0.41)^∗^
		F4 (-0.42)^∗^	CP3 (-0.40)^∗^		Cz (-0.41)^∗^
		F8 (-0.46)^∗^	CP4 (-0.41)^∗^		CP3 (-0.42)^∗^
		Fz (-0.43)^∗^			


**^∗^p < 0.05, ^∗∗^p < 0.01.**

Both groups exhibited significant negative correlations between RTs and difference-wave amplitudes. In young adults, shorter RTs were related to the higher amplitude of the LAN effect at F7, and a higher amplitude of P600a effect at C3, Cz, and CP3 in both agreement conditions. Older adults showed a different pattern. Shorter RTs were related to the higher amplitude of the LAN effect at F3 and Fz in both agreement conditions, and at F4 and F8 only in the disagree conditions. The amplitude of the P600a effect was negatively correlated only with the disagree condition at C3, CP3, and CP4 (see **Table 4**). There were no significant correlations between behavioral performance and amplitude or latency of the P600b effect.

## Discussion

This study aimed to assess aging-related changes in gender agreement processing associated with increased WM load. We compared behavioral and ERP responses of older adults with those of a group of young adults to explore the effects of WM load on the gender agreement processing in sentence comprehension.

### Behavioral Evidence

Considering that aging entails a decline in the WM system and difficulties in the syntactic processing of complex sentences (Kemper, 1986, 1987; Kemper et al., 1990, 2001), we expected that older adults would show fewer correct answers and longer RTs than the young adults. In a high WM load condition, we expected that older adults would require an increased processing cost, and this could then be easily observed in poorer subject performance.

In contrast with our hypothesis, no differences between groups were found in the percentage of correct answers in the high WM load condition. This unexpected finding may indicate that both groups showed similar difficulties in a high WM load condition. This finding may be explained by the inclusion criteria used (i.e., normal EEG and normal neuropsychological scores) because our subject selection resulted in a more homogeneous sample than previous behavioral studies (Kemper, 1986, 1987; Kemper et al., 1990, 2001). In this respect, it has been posited that EEG slowing is not necessarily a normal characteristic of aging but rather may reflect some degree of cerebral pathology (Schomer and Lopes da Silva, 2011). Therefore, by only including subjects with an EEG within normal limits of those of the same age, we may have incorporated healthier subjects into our samples. Thus, we assume that the features of our older adults are linked to a similar accuracy of responses to those of the young adult group in a high WM load condition, and as a consequence, the gender-agreement processing pattern may be behaviorally better than that reported in the literature, even when the WM load was increased.

Another fact that should be considered is that the older adults were globally slower than the young adults regardless of the WM load condition. This fact did not match with our hypothesis because we expected to find longer RTs only in the high WM load, but our older adults displayed longer RTs than young adults in both WM load conditions. Starns and Ratcliff (2010) have proposed that longer RTs could be part of a successful strategy. They have reported that older adults seem to sacrifice response speed in favor of accuracy. Although our behavioral results do not support that a high WM load has a higher cost in older than in young adults, our finding seems to be supported by this idea of implementing a successful strategy. Thus, our older adults seem to take their time to emit a more precise answer.

### ERPs Evidence

Even though the behavioral results did not completely support our hypothesis, older adults displayed different ERP amplitude modulations in a high WM load condition than young participants. In a high WM load condition, we expected that older adults would show a greater amplitude of the LAN effect, smaller amplitudes of P600a and P600b effects, and a longer P600b latency compared with young participants. In contrast, our results suggested that elderly participants were different compared with young participants in the LAN effect but showed smaller amplitudes. Older adults displayed smaller amplitudes of P600a and P600b effects in high compared with low WM load conditions, which was not observed in young participants.

Theoretically, in the first stage of the gender agreement processing (i.e., at the time window of the LAN component), lemma and grammatical features are retrieved from the lexicon. Previous evidence in young adults has shown that more words being placed between two lexical units in agreement relation (e.g., noun and adjective) may generate a greater processing cost. As they interfere in the recovering of grammatical features, this interference may be reflected in a greater amplitude of the LAN effect (Vos et al., 2001) in a higher cost condition, as was used in this experiment (high WM load condition), and thus the smaller LAN effect observed in older adults can be interpreted paradoxically as a lower processing cost. Thus, it could be interpreted as that the interference effect produced by the words placed between the noun and adjective may be less evident in older than in young adults. This fact may ideally facilitate gender agreement processing; however, it may be generated by a failure in the maintenance of word grammatical-information in the WM system.

According to previous studies in younger participants, a higher cost of processing occurs in the disagreement condition because it is necessary to identify the morpho-syntactic problem to understand the sentence. This higher difference between agreement conditions would be reflected by a larger amplitude LAN effect (i.e., the large amplitude in the difference wave). In our older adults compared with younger participants, it seems at this stage of processing that there is a reduced amplitude of LAN effect. This result may indicate that they incur a similar processing cost in the two agreement conditions (i.e., agree vs. disagree). Elderly participants also displayed a smaller amplitude of LAN effect in the high vs. the low WM load condition. This reduced amplitude of LAN effect (similar amplitudes between the agree and disagree conditions) could be due to an increase of amplitude for the agree condition or to a decrease for the disagree condition. We found that elderly participants displayed significant smaller LAN amplitudes in the disagree condition when WM load was higher, as opposed to lower. Meanwhile, in the agree condition, no amplitude differences between WM load conditions were observed. Thus, reduced amplitude of a LAN effect in the older group could be the result of a decrease of amplitude effect for the disagree condition, which may be interpreted as an incipient failure to identify the grammatical violation. Therefore, this electrophysiological pattern would suggest an age-related problem in morpho-syntactic processing when WM load is increased.

However, even when older adults seem to fail at the first stage (i.e., LAN), gender agreement processing may be conducted in later stages (Faussart et al., 1999).

In the next stage, the integration of all information associated with previous sentence context is performed (Kaan et al., 2000). Previous studies in young participants had described that a smaller amplitude of P600 effect (in this study: P600a and P600b) was observed when the WM load was increased. This amplitude decreasing has been interpreted as difficulties in agreement processing; therefore, a smaller P600 amplitude may reflect a greater processing cost (Gunter et al., 1997; Kolk et al., 2003). We expected that, when WM load increased, older adults would display a smaller amplitude of the P600a effect than younger participants. Our older adults showed the electrophysiological pattern described in previous studies (i.e., a smaller amplitude of P600a effect in the high vs. the low WM load condition). We propose that the greater processing cost observed in older adults may reflect not only the integration of all information associated with the previous sentence context but also the gender-agreement reprocessing, which could not have been completed at a previous stage (i.e., at the time-window of the LAN effect). Differences between groups are given by the fact that the groups showed opposite patterns of differences between WM load conditions. That is, older adults showed greater P600a amplitudes in the low WM load condition and in the high WM load condition for young participants.

At the last stage of the agreement processing, a generalized mapping of the sentence might be computed and this may be observed in P600b modulations (Barber and Carreiras, 2005; Molinaro et al., 2008). If there is a grammatical problem previously diagnosed within the sentence, a repair mechanism is triggered at this stage (Bornkessel and Schlesewsky, 2006). According to previous studies (Vos et al., 2001; Phillips et al., 2005), we hypothesized that older adults would show a smaller amplitude of P600b and a longer P600b latency in the high WM load condition than young adults. Our findings suggest that there are P600b amplitude differences between age groups. Specifically, older adults displayed smaller P600b amplitudes in the high, as opposed to the low WM load condition, but this pattern was not observed in young participants. A possible explanation for this finding is that older adults had to exert greater effort in repairing the sentence than did younger participants. No latency differences between groups or between WM load conditions were observed.

### Overview

The pattern of behavioral responses in the processing of gender agreement as a function of the WM load of the older adults was different from that of the younger adults, even though only their response time was significantly longer. It seems that they opted for a successful strategy of sacrificing time for precision (Starns and Ratcliff, 2010). It is likely that this strategy was useful because older adults displayed similar accuracy of response to that of younger adults, contra our expectations. The overall delay in the response times of older adults can also be expected as a natural effect of aging when solving cognitive tasks in general, regardless of the language process evaluated by the ERPs in this experiment (Wlotko et al., 2010).

The characteristic brain response pattern generated by the processing of sentence agreement (i.e., LAN, P600a, and P600b) that accounts for three consecutive stages of parsing processes (Barber and Carreiras, 2005; Molinaro et al., 2008) was evident in the older adults. However, when the WM load was increased, their agreement processing pattern was modulated differently from that of young adults.

Our results suggest that the increase in WM load causes older adults to have more failures to maintain grammatical information (i.e., in the early morpho-syntactic process) so they must carry out this process in the next phase (Faussart et al., 1999). This failure can also be evidenced by the fact that as the RTs increased the amplitude of the LAN component decreased.

In the following stages of processing, older adults must carry out all of the processing of the gender agreement. As previous studies have shown, when WM load increases, there is a higher processing cost observed in older adults with smaller amplitudes of the P600 effect (Gunter et al., 1997; Kolk et al., 2003), but they also must reprocess gender agreement when integrating the sentence arguments. This fact, which implies a high processing cost, can be interpreted from the significant differences in P600a amplitude between high and low WM load observed in older adults but not in young adults, who we suspect were able to carry out the agreement processing in the first stage.

For the last processing stage, where the complete mapping of the sentence is carried out to give it meaning, older adults also showed a higher processing cost when faced with a high rather than a low WM load. This result is supported by previous studies on the relationship between the greater load of the WM and smaller amplitude of the P600 (Gunter et al., 1997; Kolk et al., 2003). The latency in which this last stage occurs did not differ between groups, so we think that the processing cost given by the high WM load was compensated for by the number of resources invested rather than by slowing down the processing speed. The RTs recorded by the task execution do not seem to be related to the registered brain response to this last stage. This fact suggests that after processing the sentence, the participant must invest time in decision making and, of course, the motor response. Therefore, if older adults opt for a strategy of sacrificing time to improve the accuracy of their responses, it is very likely that their long RTs latencies are involved in other cognitive processes, in addition to those included in the processing of gender agreement.

### Limitations

The results of this study are not completely representative of the effects of WM in the processing of reading comprehension in aging since our results exclusively address morpho-syntactic processing. Considering that gender features of inanimate nouns are purely linguistic, and do not convey any semantic content, their processing is likely less noticeable than other types of agreement clashes (with number agreement). This could explain the unexpected behavioral results in both younger and older adults in this study and it is why we assume that the participants did not achieve as high a percentage correct as we might have expected, regardless of the group to which they belonged. Further studies could explore different kinds of agreement, including number, person or case in association with WM manipulation.

Another limitation was that the WM load seemed to impose similarly high demands on both groups, which likely reduced the expected behavioral and electrophysiological differences between them. Different levels of WM load could help to attenuate this concern.

Finally, since our study only includes older adults with normal EEG and normal neuropsychological scores, in order to confirm the behavioral findings in previous studies, further studies should also include a group of healthy older adults with abnormal EEG patterns.

## Author Contributions

GA-C, JS-P, and TF designed the research. GA-C, SC-C and JS-L acquired the data. JS-P, GA-C and JS-L analyzed the data. GA-C, JS-P, TF, MR, SC-C and JS-L contributed to data interpretation and to the writing of the paper.

## Conflict of Interest Statement

The authors declare that the research was conducted in the absence of any commercial or financial relationships that could be construed as a potential conflict of interest.

## References

[B1] Baddeley A. D. (2003). Working memory: looking back and looking forward. *Nat. Rev. Neurosci.* 4 829–839. 10.1038/nrn1201 14523382

[B2] Barber H. A. Carreiras M. (2005). Grammatical gender and number agreement in Spanish: an ERP comparison. *J. Cogn. Neurosci.* 17 137–153. 10.1162/0898929052880101 15701245

[B3] Bornkessel I. Schlesewsky M. (2006). The extended argument dependency model: a neurocognitive approach to sentence comprehension across languages. *Psychol. Rev.* 113 787–821. 10.1037/0033-295X.113.4.787 17014303

[B4] Chomsky N. (1995). *The Minimalist Program.* Cambridge, MA: MIT Press.

[B5] Chomsky N. (2001). “Derivation by Phase,” in *A Life in Language*, eds Kenstowicz M. Hale K. (Cambridge, MA: MIT Press).

[B6] Faussart C. Jacubowicz C. Costes M. (1999). Gender and number processing in spoken French and Spanish. *Riv. Linguist.* 11 75–101.

[B7] Friederici A. D. (1995). The time course of syntactic activation during language processing: a model based on neuropsychological and neurophysiological data. *Brain Lang.* 50 259–281. 10.1006/brln.1995.1048 7583190

[B8] Friederici A. D. (2002). Towards a neural basis of auditory sentence processing. *Trends Cogn. Sci.* 6 78–84. 10.1016/S1364-6613(00)01839-8 15866191

[B9] Friederici A. D. Mecklinger A. Spencer K. M. Steinhauer K. Donchin E. (2001). Syntactic parsing preferences and their on-line revisions: a Spatio-temporal analysis of event-related brain potentials. *Brain Res. Cogn. Brain Res.* 11 305–323. 10.1016/S0926-6410(00)00065-3 11275491

[B10] Gunter T. C. Stowe L. Mulder G. (1997). When syntax meets semantics. *Psychophysiology* 34 660–676. 10.1111/j.1469-8986.1997.tb02142.x 9401421

[B11] Hernández J. L. Valdés P. Biscay R. Virués T. Szava S. Bosch J. (1994). A global scale factor in brain topography. *Int. J. Neurosci.* 76 267–278. 10.3109/00207459408986009 7960483

[B12] Jackendoff R. (2007). A parallel architecture perspective on language processing. *Brain Res.* 1146 2–22. 10.1016/j.brainres.2006.08.111 17045978

[B13] Just M. A. Carpenter P. A. (1992). A capacity theory of comprehension: individual differences in working memory. *Psychol Rev.* 99 122–149. 10.1037/0033-295X.99.1.122 1546114

[B14] Kaan E. (2002). Investigating the effects of distance and number interference in processing subject-verb dependencies: an ERP study. *J. Psycholinguist. Res.* 31 165–193. 10.1023/A:1014978917769 12022794

[B15] Kaan E. Harris A. Gibon E. Holcomb P. J. (2000). The P600 as an index of syntactic integration difficulty. *Lang. Cogn. Process.* 15 159–201. 10.1080/016909600386084 15722211

[B16] Kemmer L. Coulson S. De Ochoa E. Kutas M. (2004). Syntactic processing with aging: an event-related potential study. *Psychophysiology* 41 372–384. 10.1111/1469-8986.2004.00180.x 15102122

[B17] Kemper S. (1986). Imitation of complex syntactic constructions by elderly adults. Applied ERP studies of language in aging. *Psycholinguistics* 7 277–287. 10.1017/S0142716400007578

[B18] Kemper S. (1987). Syntactic complexity and elderly adults’ prose recall. *Exp. Aging Res.* 13 47–52. 10.1080/03610738708259299 3678351

[B19] Kemper S. Rash S. Kynette D. Norman S. (1990). Telling stories: the structure of adults’ narratives. *Eur. J. Cognit. Psychol.* 2 205–228. 10.1080/09541449008406205

[B20] Kemper S. Thompson M. Marquis J. (2001). Longitudinal change in language production: effects of aging and dementia on grammatical complexity and propositional content. *Psychol. Aging* 16 600–614. 10.1037/0882-7974.16.4.600 11766915

[B21] King J. W. Just M. A. (1991). Individual differences in syntactic processing: the role of working memory. *J. Mem. Lang.* 30 580–602. 10.1016/0749-596X(91)90027-H

[B22] King J. W. Kutas M. (1995). “Do the waves begin to waver? ERP studies of language processing in the elderly,” in *Age Differences in Word and Language Processing*, eds Allen P. A. Bashor T. R. (Amsterdam: Elsevier), 314–344. 10.1016/S0166-4115(06)80077-4

[B23] Kolk H. H. Chwilla D. J. Van Herten M. Oor P. J. W. (2003). Structure and limited capacity in verbal working memory: a study with event-related potentials. *Brain Lang.* 85 1–36. 10.1016/S0093-934X(02)00548-5 12681346

[B24] Lewis R. L. Vasishth S. Van Dyke J. A. (2006). Computational principles of working memory in sentence comprehension. *Trends Cogn. Sci.* 10 447–454. 10.1016/j.tics.2006.08.007 16949330 PMC2239011

[B25] Luck S. J. (2005). *An Introduction to the Event-Related Potential Technique.* Cambridge MA: MIT Press.

[B26] McKinnon R. Osterhout L. (1996). Constraints on movement phenomena in sentence processing: evidence from event-related brain potentials. *Lang. Cogn. Process.* 11 495–523. 10.1080/016909696387132

[B27] Molinaro N. Barber H. A. Carreiras M. (2011). Grammatical agreement processing in reading: ERP findings and future directions. *Cortex* 47 908–930. 10.1016/j.cortex.2011.02.019 21458791

[B28] Molinaro N. Vespignani F. Job R. (2008). A deeper reanalysis of a superficial feature: an ERP study on agreement violations. *Brain Res.* 1228 161–176. 10.1016/j.brainres.2008.06.064 18619420

[B29] Oldfield R. C. (1971). The assessment and analysis of handedness: the Edinburgh inventory. *Neuropsychologia* 9 97–113. 10.1016/0028-3932(71)90067-4 5146491

[B30] Osterhout L. Bersick M. McLaughlin J. (1997). Brain potentials reflect violations of gender stereotypes. *Mem. Cognit.* 25 273–285. 10.3758/BF03211283 9184479

[B31] Osterhout L. Holcomb P. (1995). “Event-Related Potentials and Language,” in *Electrophysiology of the Mind: Event-Related Brain Potentials and Cognition*, eds Rugg M. Coles M. (Oxford: University Press), 171–187.

[B32] Osterhout L. Kim A. Kuperberg G. (2006). “The neurobiology of sentence comprehension,” in *The Cambridge Handbook of Psycholinguistics*, eds Spivey M. Joannisse M. Macrae K. (Cambridge, MA: Cambridge University Press).

[B33] Osterhout L. McKinnon R. Bersick M. Corey V. (1996). On the language – specificity of the brain response to syntactic anomalies: Is the syntactic positive shift a member of the P300 family? *J. Cogn. Neurosci.* 8 507–526. 10.1162/jocn.1996.8.6.507 23961982

[B34] Osterhout L. McLaughlin J. Allen M. Inoue K. (2002). Brain potentials elicited by prose-embedded linguistic anomalies. *Mem. Cognit.* 30 1304–1312. 10.3758/BF03213412 12661861

[B35] Osterhout L. Mobley L. A. (1995). Event-related brain potentials elicited by failure to agree. *J. Mem. Lang.* 34 739–773. 10.1006/jmla.1995.1033

[B36] Osterhout L. Nicol J. (1999). On the distinctiveness, independence, and time course of the brain responses to syntactic and semantic anomalies. *Lang. Cogn. Process.* 14 283–317. 10.1080/016909699386310

[B37] Park D. C. Lautenschlager G. Hedden T. Davidson N. S. Smith A. D. Smith P. K. (2002). Models of visuospatial and verbal memory across the adult life span. *Psychol. Aging* 17 299–320. 10.1037/0882-7974.17.2.299 12061414

[B38] Phillips C. Kazanina N. Abada S. H. (2005). ERP effects of the processing of syntactic long-distance dependencies. *Brain Res. Cogn. Brain Res.* 22 407–428. 10.1016/j.cogbrainres.2004.09.012 15722211

[B39] Reisberg B. Ferris S. H. De Leon M. J. Crook T. (1982). The global deterioration scale for assessment of primary degenerative dementia. *Am. J. Psychiatry* 139 1136–1139. 10.1176/ajp.139.9.1136 7114305

[B40] Schomer D. L. Lopes da Silva F. H. (2011). *Niedermeyer’s Electroencephalography, Basic Principles, Clinical Applications and Related Fields*, 6th Edn Philadelphia, PA: Lippincott Williams & Wilkins.

[B41] Silva-Pereyra J. Carreiras M. (2007). An ERP study of agreement features in Spanish. *Brain Res.* 1185 201–211. 10.1016/j.brainres.2007.09.029 17963736

[B42] Silva-Pereyra J. Rodríguez-Camacho M. Prieto-Corona B. Aubert E. (2014). *LEXMEX. Diccionario de frecuencias del español de México.* México: Facultad de Estudios Superiores Iztacala, UNAM.

[B43] Starns J. J. Ratcliff R. (2010). The effects of aging on the speed-accuracy compromise: boundary optimality in the diffusion model. *Psychol. Aging* 25 377–390. 10.1037/a0018022 20545422 PMC2896207

[B44] Szava S. Valdes P. Biscay R. Galan L. Bosch J. Clark I. (1994). High resolution quantitative EEG analysis. *Brain Topogr.* 6 211–219. 10.1007/BF01187711 8204408

[B45] Valdés P. Biscay R. Galán L. Bosch J. Zsava S. Virués T. (1990). High-resolution spectral EEG norms topography. *Brain Topogr.* 6 281–282.

[B46] Van Dyke J. A. (2007). Interference effects from grammatically unavailable constituents during sentence processing. *J. Exp. Psychol. Learn. Mem. Cogn.* 33 407–430. 10.1037/0278-7393.33.2.407 17352621 PMC2077343

[B47] Van Dyke J. A. Johns C. L. (2012). Memory interference as a determinant of language comprehension. *Lang. Linguist. Compass* 6 193–211. 10.1002/Inc3-330 22773927 PMC3389825

[B48] Vos S. H. Gunter T. C. Kolk H. H. Mulder G. (2001). Working memory constraints on syntactic processing: an electrophysiological investigation. *Psychophysiology* 38 41–63. 10.1111/1469-8986.3810041 11321620

[B49] Waters G. S. Caplan D. (1996). The capacity theory of sentence comprehension: critique of Just and Carpenter (1992). *Psychol. Rev.* 103 761–772. 10.1037/0033-295X.103.4.761 8888653

[B50] Wechsler D. (2003). *Escala Wechsler de Inteligencia para Adultos III, Manual de técnico.* México: Manual Moderno.

[B51] Wlotko E. W. Chia-Lin L. Federmeier K. D. (2010). The language of the aging brain. Event-related potential studies of comprehension in older adults. *Lang. Linguist. Compass* 4 623–638. 10.1111/j.1749-818X.2010.00224.x 20823949 PMC2930790

[B52] Zar J. H. (1998). *Biostatistical Analysis*, 4th Edn Englewood Cliffs, NJ: Prentice Hall.

